# Emotional representations of space vary as a function of peoples’ affect and interoceptive sensibility

**DOI:** 10.1038/s41598-021-95081-9

**Published:** 2021-08-09

**Authors:** Alejandro Galvez-Pol, Marcos Nadal, James M. Kilner

**Affiliations:** 1 Balearic Islands Health Research Institute (IdISBa) and Research Institute of Health Sciences (IUNICS) , Palma de Mallorca, Spain; 2 Human Evolution and Cognition Research Group (EvoCog), Psychology Dept. University of the Balearic Islands, Palma de Mallorca, 07122 Spain; 3 Dept. of Clinical and Movement Neurosciences, Queen Square Institute of Neurology, University College London, London, WC1N 3BG UK

**Keywords:** Psychology, Human behaviour, Psychology and behaviour

## Abstract

Most research on people’s representation of space has focused on spatial appraisal and navigation. But there is more to space besides navigation and assessment: people have different emotional experiences at different places, which create emotionally tinged representations of space. Little is known about the emotional representation of space and the factors that shape it. The purpose of this study was to develop a graphic methodology to study the emotional representation of space and some of the environmental features (non-natural vs. natural) and personal features (affective state and interoceptive sensibility) that modulate it. We gave participants blank maps of the region where they lived and asked them to apply shade where they had happy/sad memories, and where they wanted to go after Covid-19 lockdown. Participants also completed self-reports on affective state and interoceptive sensibility. By adapting methods for analyzing neuroimaging data, we examined shaded pixels to quantify where and how strong emotions are represented in space. The results revealed that happy memories were consistently associated with similar spatial locations. Yet, this mapping response varied as a function of participants’ affective state and interoceptive sensibility. Certain regions were associated with happier memories in participants whose affective state was more positive and interoceptive sensibility was higher. The maps of happy memories, desired locations to visit after lockdown, and regions where participants recalled happier memories as a function of positive affect and interoceptive sensibility overlayed significantly with natural environments. These results suggest that people’s emotional representations of their environment are shaped by the naturalness of places, and by their affective state and interoceptive sensibility.

## Introduction

Our diverse activities commonly require us to move from one place to another^1–3^. We find our way from one place to the next using complex spatial representations, and we decide where to go and how long to stay there based on appraisals and expectations. Most of what is known about people’s representation of space comes from studies on spatial navigation and appraisal^1–8^. But the places that make up our environment are not only places we go to and come from; they are also the stages upon which we have experiences, upon which we experience our own bodies. Research on spatial representation has commonly overlooked two crucial facts. First, people’s representations of space are emotionally tinged. Second, people’s representations of space are based on internal models of their own bodies.

### Representations of space are emotionally tinged

As we move through the environments, our representation of space (i.e., cognitive maps^2,9^) becomes intertwined with emotional experiences. Some of these experiences might be pleasant, others unpleasant, and others indifferent. We might wish for more of such experiences, or long for different ones. As a result, our cognitive maps get updated with both past emotional experiences and desired future states^7,10,11^. For instance, we might feel excited or happy as we stroll along a beach or hike up a mountain, whereas during the Covid-19 pandemic lockdowns we might feel cut off at home, and eager for the seaside or the mountains.

### Representations of space are based on internal body models

Recent evidence shows that people’s assessment of their environment involves running an internal model of their body in the world^12,13^. Such a model reflects information about their affective state, physiological state, and desired future states^12–15^. This idea is supported by studies that show how differences in the way people feel and appraise their inner bodily states (i.e., affective state and interoception, respectively) influence how they relate to features of their environment^16–21^. Thus, crucial to the representation of space is the weighing of bodily representations against the computed value of certain environmental features^22,23^. In this context, people appraise and seek out certain environments because of the relevant resources for psychophysiological balance they afford^24^. This balance is achieved through the constant regulation and anticipation of future needs and challenges in the environment (i.e., allostasis)^25,26^.

**Figure 1:**
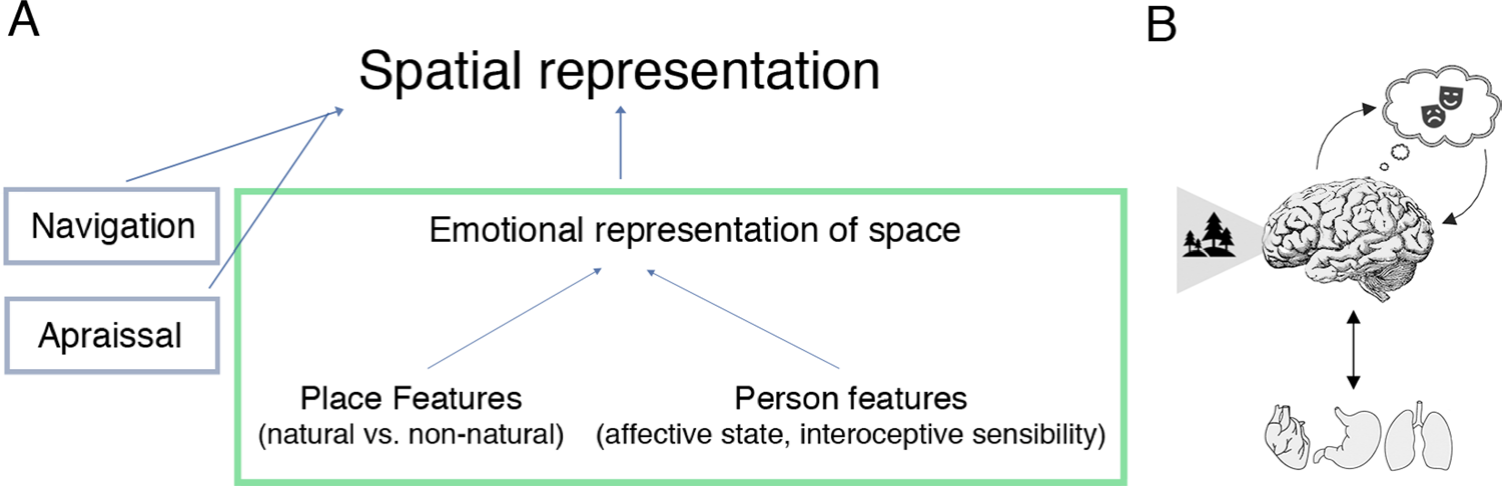
Framework and conceptualization. (**A**) People’s representation of space includes aspects related to navigation and appraisal. Yet, it is often overlooked that people have emotional experiences at different places, which create emotionally tinged representations of space. The current work (green box) examined these by considering that emotional representations are modulated by person and place features. The variables examined here (affective state, interoceptive sensibility, and natural vs. non-natural locations) reflect current models of human cognition. (**B**) Under these models, people’s emotional representations are accomplished by weighting bodily representations against value computations, namely, how we feel (based on past experiences and predicted needs) and what the environment can provide^12–14^. Icons in panel (**B**) are under Creative Commons license CC0 from Pixabay.com.

In sum, people’s representation of space includes aspects related to navigation and appraisal but, crucially, also aspects related to emotion: the emotional representation of space. The main purpose of this study was to examine people’s emotional representation of space and how it is shaped by certain environmental features, such as whether a place is natural or non-natural, and certain personal features, such as affective state and interoceptive sensibility (Fig. 1).

The current study was conducted during the Spanish nationwide Covid-19 lockdown (March-June 2020) and restricted to the island of Mallorca, Spain (~ 100 km wide × 75 km long). This allowed us to examine past emotional experiences in space and desire for future environments after a period of distress and restricted mobility. The target territory, bounded by the sea, included a UNESCO World Heritage cultural site (where the natural landscape merges with small villages), fields, towns, crops, and cities, providing a diverse testing ground to examine our hypotheses.

**Figure 2:**
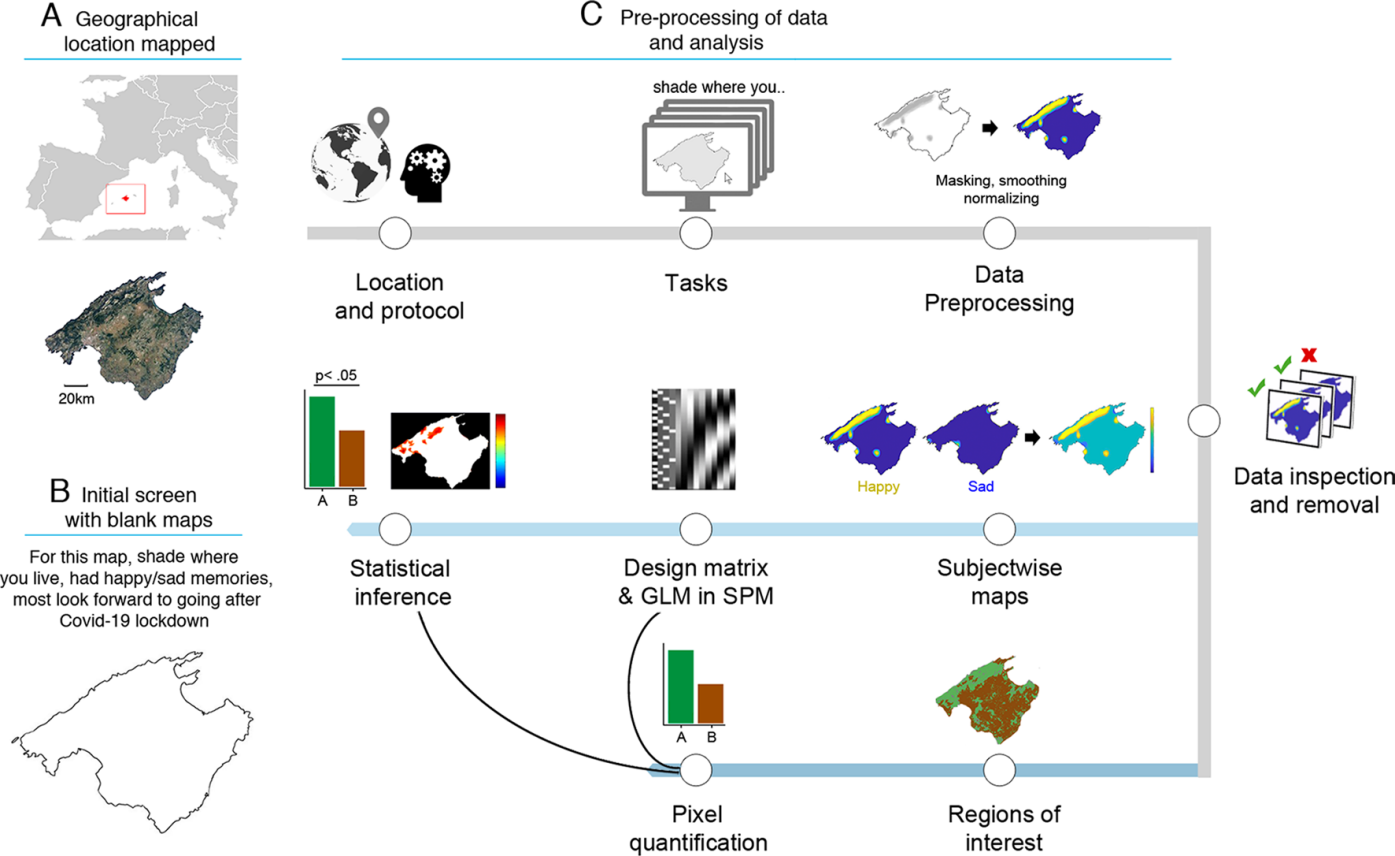
Task and processing of data. (**A**) Location of mapped territory: Mallorca island, Spain, 39.6953° N, 3.0176° E. (**B**) Task: participants performed a computer-based task in which they shaded four separate maps (remotely) to indicate where they lived, where they had happy memories, where they had sad memories, and where they most looked forward to going after the Spanish national Covid-19 lockdown. Also, they previously filled in two self-report questionnaires measuring affect and interoceptive sensibility. (**C**) Data preprocessing and analysis: maps were saved as matrices, masked, smoothed, and normalized. After data inspection, the happy and sad memory maps were combined into subject-wise maps and subjected to Statistical Parametric Mapping (i.e., to examine whether the mapping response was similar across participants and locations). Next, we quantified the proportion of pixels overlapping with natural and non-natural environments (regions of interest). Also, the subject-wise maps were correlated with the scores of the self-reports. Regarding the Covid-19 lockdown map, we only quantified the proportion of pixels in natural and non-natural environments. Outline maps are under Creative Commons license CC0 from Pixabay.com.

To achieve our goal, we developed a new approach that integrated the recollection of autobiographical memories, self-report questionnaires, and graphic methods adapted from those used to analyze neuroimaging data (Fig. 2). Participants were asked to perform a computerized task consisting in shading four separate blank maps of Mallorca to show where they lived, where they had happy memories, where they had sad memories, and where they most looked forward to going after the Spanish national Covid-19 lockdown. This task required participants to access autobiographical memories, which are known to encompass past emotional experiences^27,28^. Importantly, their valence can be assessed by asking participants to associate these memories with ‘basic’ emotions^28–32^. Next, we examined how the representation of these emotional memories in space were shaped by the two person features noted above: affective state and interoceptive sensibility. To this end, we recorded participants’ scores in two self-report questionnaires: the Depression, Anxiety, and Stress scale (DASS21)^33^, which measures affective state^34^ in the form of scores in depression, anxiety, and stress, and the Multidimensional Assessment of Interoceptive Awareness (MAIA)^35^, which measures the capability to appraise inner bodily sensations (currently termed interoceptive sensibility)^36^. Factoring these measures into the analysis would us to examine whether the representation of emotional memories in space varied according to individual differences in affective state and the conscious processing of inner bodily states.

As noted above, we also wished to examine how place features contribute to shaping emotional representations of space. To this end, we contrasted the participants’ shaded areas with an objective representation of natural and non-natural environments. This allowed us to test the overlapping of their responses in one *vs.* the other environment (Fig. 2) and to contextualize our results within a growing number of studies that have compared peoples’ affect and physiological regulation in natural and urban locations^37–42^. Finally, given that emotional representations of space might be influenced by previous allostasis (our past experiences determine our present concerns, which in turn influence prospective future behaviours^12,26^), we asked our participants to represent those locations that they most looked forward to going after the national Covid-19 lockdown. Then, we contrasted again these responses with an objective representation of natural and non-natural environments (see “Methods” section; Table 1).

**Table 1:** Summary of data collected, measures, and framework.

Data collected	Measures	Framework
Spatial maps of emotional memories (past experiences)	Distribution of participants’ happy and sad memories in topographical space (emotional memories); Statistical Parametric Mapping of pixels	﻿Maps based on participants’ autobiographical memories. Included to better understand how people perceive their environment based on past experiences
Spatial maps of desired locations (future behaviour)	Distribution of participants’ desired locations in topographical space; Statistical Parametric Mapping of pixels	Maps based on participants’ desired location (after nationwide Covid-19 lockdown). Included to examine prospective behaviour based on preferred environments
Multidimensional Assessment of Interoceptive Awareness (MAIA)	Self-report with 8 scales: capability to notice, attend, regulate, listen, connect, trust bodily sensations, and to ignore sensations of pain or discomfort; 6-point Likert scale from 0 (never) to 5 (always). Scores calculated individually by adding up the values of participants’ responses	Measuring the capability to appraise and use inner bodily sensations; tightly link to physiological underpinning of human emotions. The name of the questionnaire indicates that it measures ‘interoceptive awareness’, but it is now accepted that it measures interoceptive sensibility. See Ref.^36^
The Depression, Anxiety, and Stress scale (DASS21)	Self-report with 3 subscales (7 items each) scoring participants’ negative affect on a 4-point Likert scale from 0 (did not apply to me at all) to 3 (applied to me very much, or most of the time). Scores calculated individually by adding up the values of participants’ responses	﻿Measuring 3 related negative emotional states (negative affect; see *note*). Together with MAIA data, this allows to correlate felt psychophysiological differences with the above mapping responses

Significant clusters in the maps were revealed when participants reported similar emotional memories, in similar regions, and in a similar manner (happy or sad); Participants’ maps were also correlated with their scores in the MAIA and DASS21, revealing locations distinctively recalled as function of participants’ felt psychophysiological differences; Spatial maps of emotional memories and desired locations were also contrasted with an objective map of natural and non-natural environments, allowing to test the overlapping of the mapping response in one *vs.* the other environment; The term ‘affect’ is used in the manuscript to refer to the scores of the DASS21, which is intended to measure negative affect^17,18^.

Each of the four shaded maps produced by each of the participants was subjected to Statistical Parametric Mapping (SPM12^43^) a statical technique used to examine brain activity recorded in functional neuroimaging experiments (Fig. 2). We examined shaded pixels instead of voxels in the brain to quantify where in the maps and how strong participants’ responses relate to one’s geographical space (i.e., are certain locations consistently associated with particular emotions?). This approach does not rely on the semantic report of the participants (i.e., opinions about specific points in space), which might create problems for the statistical interpretation of the data, such as uneven information across participants or abstract judgments^4^ (see “Methods”). Also, it allows using the same method across different populations and target environments, as well as contrasting the subjective response of the population with more objective data (e.g., maps with environmental features).

Given the relatively small size of the island where the sample was taken and the study performed, and that cognitive maps function as shared repositories for personal and group information about places, relationships, beliefs, and values^44^, we hypothesized that participants would convergently shade in the same areas. Yet, we also postulated significant differences in the mapping response according to individual psychophysiological differences. Specifically, we hypothesized that certain locations would be associated with happier memories when accompanied by more positive affect and higher interoceptive sensibility. Moreover, considering that experiences in natural areas have been linked to improved mental health and physiological regulation^37–42^, we hypothesized that these regions and desired locations after the lockdown would be associated with natural environments.

## Results

### Spatial maps of emotional memories, environmental features, and individual differences in affect and interoceptive sensibility

**Figure 3:**
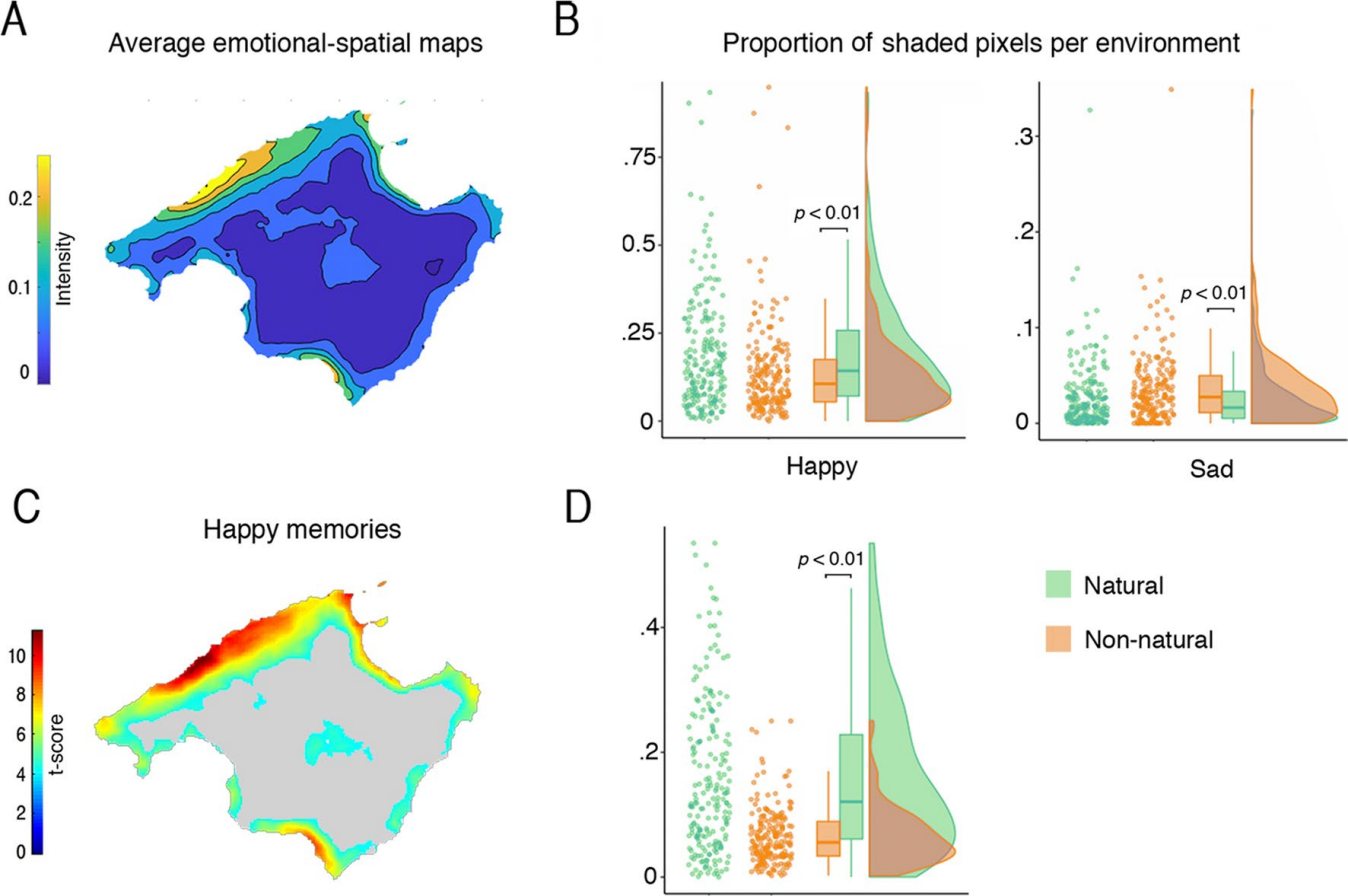
Spatial maps of emotional memories. (**A**) Subject-wise emotional-spatial maps that combine happy and sad memory maps. The colorbar denotes participants’ mean intensity response (warmer colors for happier memories in space). (**B**) We quantified the proportion of pixels that participants shaded for each environment (according to the total number of pixels in natural or non-natural environments), and the proportion of these shaded regions for each emotion. Here, a significant interaction with follow-up analysis showed that happy memories occurred more in natural environments and vice versa (p < 0.001). (**C**) SPM analyses of the subject-wise emotional-spatial maps showed significant areas only for happy memories (i.e., consistent mapping significantly > 0; FWE corr.). (**D**) We also quantified the number of pixels per environment in the significant SPM clusters of panel (**C**). The number of pixels in natural environments was greater than in non-natural (*p* < 0.01); bars denote the t-statistic range; n = 201; comparisons between environments computed via Wilcoxon’s statistical test. Outline maps are under Creative Commons license CC0 from Pixabay.com.

We asked our participants to shade the maps where they have happy and sad memories. The mean proportion of shaded territory was 0.18 (*SD* = 0.15) for happy memories and 0.05 (*SD* = 0.04) for sad memories. The subject-wise combination of happy and sad maps yielded a mean proportion of shaded territory of 0.20 (*SD* = 0.15). Figure 3A shows participants’ mean map. Next, for the maps that participants shaded, we estimated the proportion of shaded pixels that overlapped with natural and non-natural environments. The latter included human-made locations such as cities or towns, and non-accessible human-altered environments such as crop fields and cattle raising areas (see “Methods” and Supp. materials). To this end, we used publicly available government forest maps, computed the proportion of shaded pixels that overlapped with each environment, and normalized these proportions according to all the pixels available per environment. Then, we computed these proportions in a repeated-measures ANOVA with two factors and two levels: environment (natural, non-natural) and Emotional memory (happy, sad). This analysis yielded significant main effects of Environment (*F*_(1,200)_ = 33.55, *p* < 0.001, *η*_*p*_^2^ = 0.144) and Emotional memory (*F*_(1,200)_ = 165.45, *p* < 0.001, *η*_*p*_^2^ = 0.453), and a significant interaction between them (*F*_(1,200)_ = 69.81, *p* < 0.001, *η*_*p*_^2^ = 0.259). We followed up this interaction by comparing the proportion of pixels reported for happy and sad emotional memories in each environment. This analysis revealed that the proportion of pixels were participants reported happy memories in natural areas (*Mdn* = 0.143), was significantly higher (*Z* = 5230, *p* = < 0.001, *r* = 0.480) than in non-natural areas (*Mdn* = 0.106). Conversely, the proportion of pixels reported for sad memories in natural areas (*Mdn* = 0.016) was significantly lower (*Z* = 14,522, *p* = < 0.001, *r* = 0.551) than in non-natural areas (*Mdn* = 0.027); Fig. 3B.

After the pixel quantification, we examined whether emotional memories can be consistently mapped in geographical space. We subjected the subject-wise maps to Statistical Parametric Mapping^43^. The results showed that participants’ emotional memories consistently relate to locations in space: there was an overlap in areas shaded by participants (across participants, same locations shaded with similar valence). Yet, this was only observed for places where they reported having happy memories (i.e., consistent mapping significantly > 0; FWE corr. Fig. 3C,D). Places where participants reported having negative memories were not consistently mapped. Alternatively, ambivalent places that were marked as both happy and sad could have cancelled each other out.

**Figure 4:**
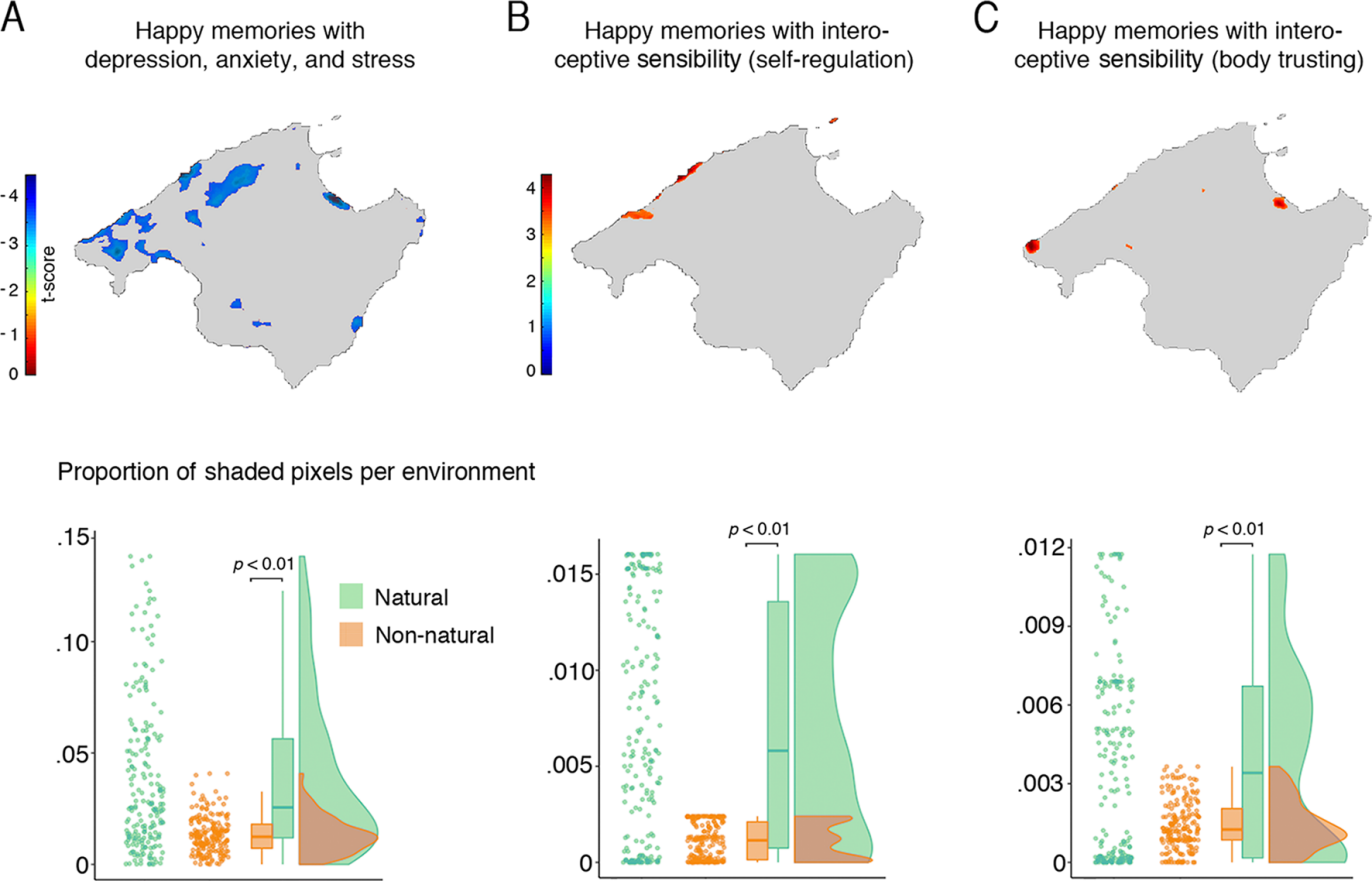
Spatial maps of emotional memories correlate with affect and interoceptive sensibility. SPM analyses of the subject-wise maps showed that spatial maps of emotional memories correlated with participants’ scores in the Dass21 and two subscales of the MAIA questionnaire. (**A**) Areas where participants with lower scores in depression, anxiety, and stress (Dass21) reported having happier memories (uncorr. at 0.01). In these clusters, the number of pixels in natural environments was greater than in non-natural (lower panel, *p* < 0.001). (**B**,**C**) Areas where participants with greater interoceptive sensibility (self-regulation and Body trusting subscales) reported having happier memories (uncorr. at 0.01). In all these SPM clusters, the number of pixels in natural environments was greater than in non-natural (*p* < 0.001); n = 201; colorbars denote the t-statistic range; comparisons between environments computed via Wilcoxon statistical test. Outline maps are under Creative Commons license CC0 from Pixabay.com.

We also examined the relationship between participants’ affective state and emotional representations of space by correlating their scores in the Dass21 questionnaire (Spanish validated version^45^) with their spatial maps of emotional memories. This revealed that several areas correlated negatively with the Dass21, meaning that participants whose affective state was more positive consistently reported having happier memories at these locations (Fig. 4A). Also, we assessed the correlation of each subscale of the Dass21 questionnaire (i.e., depression, anxiety, and stress scales) with the participants’ spatial maps of emotional memories. This revealed smaller regional clusters that negatively correlated with each subscale (Supp. Figure 3).

Likewise, we also examined the relationship between participants’ interoceptive sensibility and their emotional representation of space by correlating their scores in the MAIA questionnaire (Spanish validated version^46^) and their spatial maps of emotional memories. This allowed examining whether the sensing and appraisal of inner bodily sensations, a key constituent of one’s cognitive and affective experience, relates to one’s emotional memories. The results showed that two dimensions of interoceptive sensibility correlated positively with certain areas of the map. These were Self-Regulation and Trusting of the body, namely, one’s ability to regulate distress by attending to bodily sensation and to experience one’s body as safe and trustworthy^35^. These areas were linked to happier memories when accompanied by high scores in these dimensions of interoceptive sensibility (Fig. 4B,C). Additionally, we inspected the proportion of pixels corresponding to natural and non-natural environments in the areas revealed by the SPM analysis. To this end, we computed the environment where participants reported happy memories, areas that negatively correlated with higher scores on the DASS scale, and areas that positively correlated with higher interoceptive sensibility. In all these areas, the proportion of reported pixels was greater for natural than for non-natural areas (all *P*s < 0.01; Fig. 4A–C; see Supp. Figure 4 for the overlap between these areas and circumscribed—protected natural spaces).

Overall, our results show that people’s spatial maps of emotional memories relate to geographical areas in a consistent manner, yet this was only observed for places where they reported having happy memories. No consistent significant mapping (or additional correlations) was found for sad memories in space. In addition, our results show that certain regions were correlated with participants’ happy memories when they scored lower in negative affect and higher in interoceptive sensibility.

### Preference for visiting natural environments after the Covid-19 lockdown

**Figure 5:**
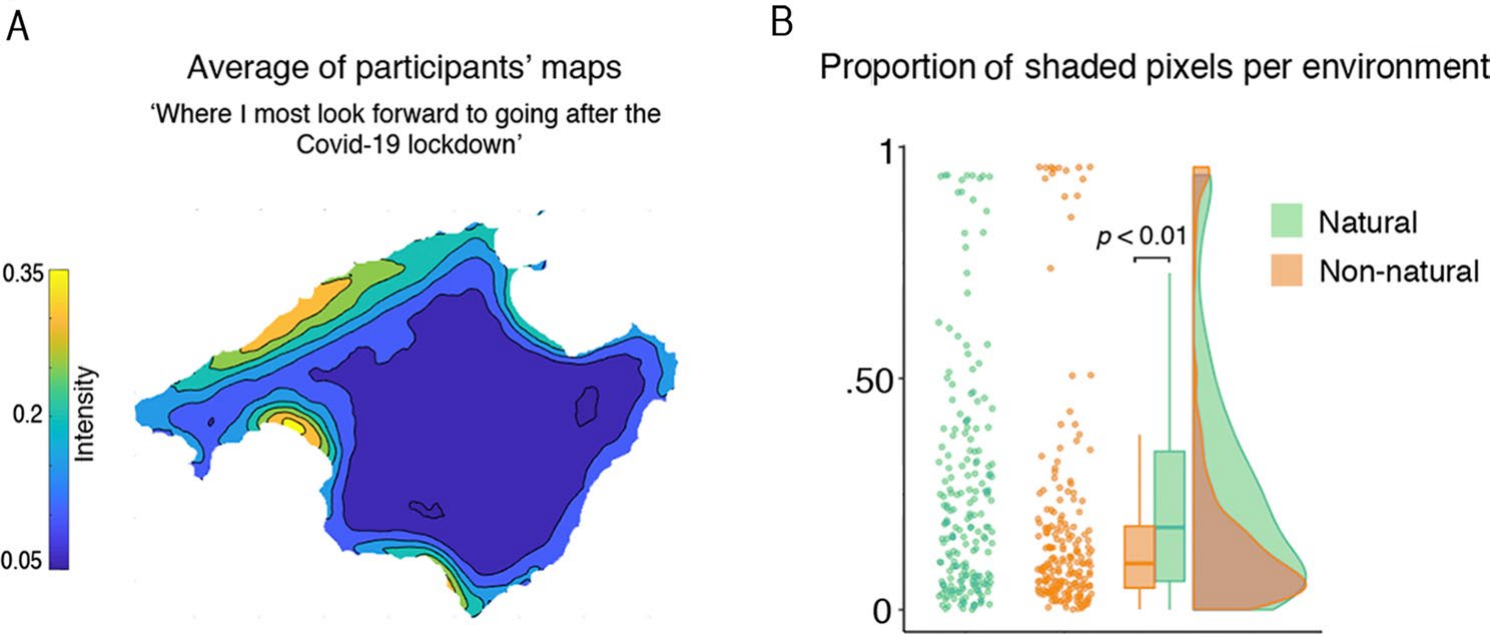
Preference for the environment after Covid-19 lockdown. (**A**) The map depicts the participants’ mean preference for regions to visit after the lockdown. The colorbar denotes the participants’ mean intensity response (warmer coloration for more desired areas). (**B**) The quantification of pixels per environment was computed via Wilcoxon statistical test. This showed that participants’ preference was significantly greater for natural than for non-natural environments (*p* < 0.001); n = 201. Outline maps is under Creative Commons license CC0 from Pixabay.com.

Studies on the psychological effects of the pandemic have revealed an increase in mental illness, somatization, and drug abuse^47,48^. Given that certain environments may feature relevant resources for the attainment of psychological and physiological balance (allostasis), we also asked our participants to report where they most looked forward to going after the nationwide Covid-19 lockdown. The mean proportion of colored territory in these maps was 0.22 (SD = 0.24); see participants’ mean map in Fig. 5. For this data, we calculated which pixels of the map corresponded to natural *vs.* non-natural areas. The analysis revealed that the proportion of pixels were participants wanted to go after the lockdown was significantly greater for natural (*Mdn* = 0.178) than for non-natural environments (*Mdn* = 0.010), *Z* = 4043, *p* = < 0.0001, *r* = 0.602. In line with previous studies^41,42,49^, our results suggest that natural environments might provide a key to palliate present and forthcoming psychological distress by aiding to attain one psychophysiological equilibrium. This relationship is also suggested in an exploratory analysis where we related the proportion of pixels per desired environment with participants’ affect and interoceptive sensibility (see Suppl. analysis and Supp. Figure 5).

### Living location does not moderate peoples’ environmental preference

Based on the participants’ living location map, we computed whether they lived in a natural or non-natural location. Next, we added the factor Living Location back to the initial quantification of shaded pixels in the subject-wise maps of emotional memories (rmANOVA with factors Environment and Emotional memory). The following interaction was not significant (*F*_(1,199)_ = 0.891, *p* = 0.346, *η*_*p*_^2^ = 0.004), suggesting that participants’ living environment did not modulate their mapping response in environmental space.

Furthermore, we observed that many participants lived in urban regions (81%). It follows that many participants could have reported both happy and sad memories in urban areas because they lived there. Considering that shading the same area as happy and sad might cancel the values of the subsequent region (i.e., ambivalent area; after combining happy and sad memory maps), participants’ living location could bias our results by cancelling out more non-natural than natural areas. Therefore, we decided to repeat our initial rmANOVA (Environment x Emotional memory) without combining happy and sad memory maps. The subsequent analyses yielded similar results. A significant interaction and main effect with more happy memories in natural environments (all *p*s < 0.01). Likewise, we computed the same rmANOVA in the subject-wise emotional memory maps by mitigating the effect of one’s living location. To do this, we subtracted the participants’ living location maps from their subject-wise emotional maps, and renormalized the proportion of coloured areas per environment on an individual basis. After implementing this step, the results remained the same: happy memories were more frequent in natural areas (all *p*s < 0.01). Lastly, we also related participants’ living location and where they wanted to go after the nationwide lockdown. The results showed that participants’ living location did not predict their environmental preference after the lockdown (*F*_(1,199)_ = 0.034, *p* = 0.855), R^2^ = -0.005; see Suppl. Figure 6.

### Participants’ affect correlates with interoceptive sensibility

**Figure 6:**
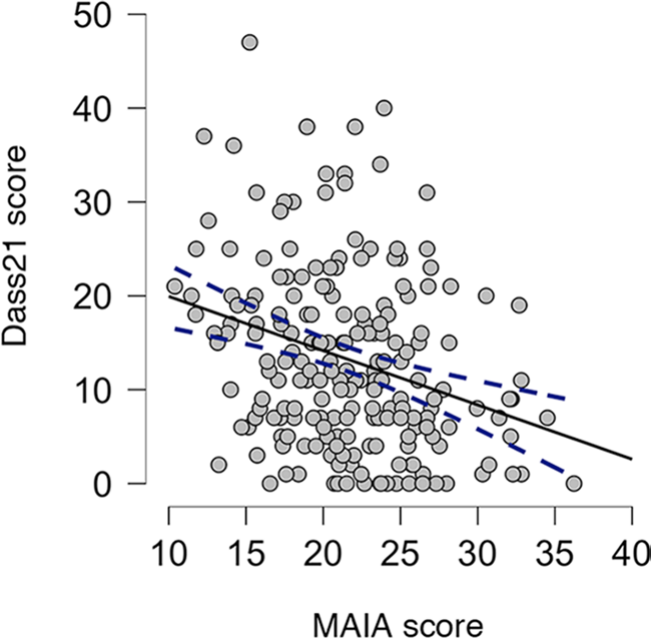
Negative correlation between affect and interoceptive sensibility. Spearman’s rho revealed a significant correlation between the participants’ Dass21 and MAIA scores, i.e., lower levels of affect were negatively correlated with higher levels of interoceptive sensibility. Dashed lines denote confidence interval; n = 201.

As measured by the Dass21 and MAIA questionnaires^33,35^, we observed that participants affect correlated with their capability to notice and appraise inner bodily sensations (i.e., interoceptive sensibility). Specifically, participants with lower scores in the Dass21 questionnaire also scored higher in interoceptive sensibility (Spearman’s rho = − 0.281, *p* = 5.467e−5, Fig. 6; see Suppl. Figure 7 for subscales correlations). Previous research that used the same self-report questionnaires reported a similar association between interoceptive sensibility, depression, anxiety and stress^50,51^. Specifically, higher scores on the Dass21 significantly correlated with lower scores on the MAIA. Consistent with such research, we found similar results but in a larger sample. These findings support the validity of our self-report data with those of previous studies.

## Discussion

The representation of space is embedded with memories of experiences and emotions. Yet, most studies on the representation of space have focused on navigational behaviors or judgements about locations. Moreover, when relating people’s affective and overall experience in space, the current methods often rely on the participants’ position during the response time (e.g. linking self-reports and GPS coordinates/zip codes^38,52^) or semantic reports^4,5^. However, mobility across the geography and how we relate to our territory are complex dynamics that might be difficult to measure with such methods. For instance, we might feel and reason about our environment even in the absence of actual mobility (during the nationwide Covid-19 lockdown). Here we developed a new paradigm that even in this case allowed us to sample the relationship between the emotional representation of one’s space, types of environment, affect, and interoception.

We gave participants blank maps of the region where they lived and asked them to shade the regions where they had happy and sad memories, as well as where they most looked forward to going after the Covid-19 national lockdown (past emotional experience and future desired locations in space). They also filled in two self-report questionnaires about their affect and capability to appraise inner bodily sensations (i.e., interoceptive sensibility). Based on techniques for neuroimaging, we analyzed shaded pixels in the maps to reveal the consistency of participants’ shading in the maps (i.e., whether participants shaded in similar regions with similar values), the correlation of this shading with their scores in the self-reports, as well as the proportion of shaded pixels overlapping with natural and non-natural environments. Our results showed that (1) happy memories are consistently mapped in geographical space; (2) that participants’ mapping seems to vary according to how they feel (affect) and interoceptive sensibility; (3) that areas reflecting positive memories overlapped more with natural than non-natural environments; (4) and that after the nationwide Covid-19 lockdown, participants wanted to visit more those areas that also corresponded to natural environments. Accordingly, we highlight three aspects of this work that need to be considered: its biological significance, the possibility of applying our results to other territories, and the flexibility of our method to sample further person-space interactions.

### Biological significance

Participants’ mapping of emotions revealed that certain areas were associated with positive memories, more positive affect, self-regulation, and trusting in bodily sensations. These later aspects refer to one’s ability to regulate distress by attending to bodily sensations and to experience one’s own body as safe. Research on these aspects often relates individual differences to psychophysiological variables^53–57^. However, how these aspects can be mapped across people’s surroundings has not been examined. In our study, natural environments overlapped most with participants’ happy memories in space, lower negative affect, and higher interoceptive sensibility. Based on this and the notion of allostasis (i.e., keeping psychophysiological stability through behavioural change^25^), we suggest there is an interaction between environment, stressor exposure, mental health, and interoceptive sensibility. These results are in line with studies showing that natural spaces constitute a fundamental resource for mental health and physiological engagement through cardiovascular exercise^39,41,58–62^. In this context, past experiences in certain areas could exert a more restorative effect upon participants’ affect (i.e., lessening levels of depression, anxiety and stress). For instance, green and blue spaces have been associated with less psychological stress and rumination^37,39^. Additionally, after the national Covid-19 lockdown, participants wanted to visit areas that corresponded to natural environments (as prospective behaviour).

We believe that natural environments were chosen because they could offer psychophysiological alleviation^38,41^ and not because these spaces were more suitable during a pandemic. The study was conducted after the very first month of the national lockdown, therefore, it is unlikely that our participants had enough knowledge about the spread of the virus and the upcoming policies about spatial restrictions. An alternative explanation, which does not exclude the former, is that people with better affect might benefit more from these environments. In addition, we found that happy memories in space correlated with two subscales of the MAIA questionnaire: self-regulation and Body trusting. Considering that this mapping response overlapped the most with natural environments, it is reasonable to think that certain natural environments require that people ‘trust their bodies’ to positively experience the environment. One aspect that needs further examination is the direction of this relationship (i.e., do people increase their interoceptive sensibility by experiencing natural environments? is that people with higher scores in these subscales who attend/experience these environments the most?). Further research could examine these novel observations by carrying on longitudinal studies and pre- and post- interventions with natural environments.

### Generalization of our results

We tested our method in an environment that ranged from fully natural (e.g., UNESCO World Heritage site) to fully urbanized areas. Considering that numerous studies show that natural spaces promote health by providing psychophysiological alleviation^38,41,42,61^, we believe that our results can generalize beyond the idiosyncrasy of the mapped territory. In addition, we update previous accounts by suggesting that even past experiences in natural environments might aid mental health under challenging circumstances. This is indicated by our finding that certain natural locations reflect past positive experiences when accompanied by lower negative affect (as measured during the Covid-19 lockdown with the self-report Dass21). Interestingly, our participants reported comparable scores in this self-report (*Ms* = 6.3, 4, and 3 in depression, anxiety, and stress; normal severity) than those found in another study with a similar population and testing period^48^.

### Methodological considerations

Previous studies have already mapped feelings and judgments in space, for instance by combining public participation and Information System Mapping^5,6,8,63^. This approach usually collects participants’ commentaries and opinions about specific points in space. Although of considerable interest, the use of semantic reports might face various challenges for the statistical interpretation of the data, such as the uneven distribution of information within and between participants’ reports (e.g., semantically uncertain and abstract judgments about places)^4^. In this context, while our method does not consider individual accounts, it does not rely on the semantic variability and interpretation of participants’ reports. Altogether, we believe that our work offers one approach to better understand human behaviour in its ecological niche, which should be complemented with methods as the ones mentioned above. Relatedly, our work is inspired by the field of neuroimaging, which started developing in the 90s. From our standpoint, what we offer here is just a glimpse of this development, i.e., a standard and relatively simple way to analyse spatially distributed data (and correlates with questionnaires). Nevertheless, we hope that future experimental studies will generate novel findings by using this or more complete approaches that could stem from the crossing between different fields.

On a different note, the spatial resolution of our method can vary according to the scope and sampled territory. In our maps, we sampled the territory with relatively low spatial resolution. Specifically, each pixel represented approximately ~ 0.06 km^2^ of the real land (~ the size of a Polo field), whereas it is likely that mapping smaller environments could lead to resolutions of square dekameter or meter. In addition, our method can be easily implemented to sample human cognition in a range of spatially extended environments (e.g., museums, towns, cities, countries, natural reserves). By doing so, not only basic science but also more practical matters such as the design, development, and allocation of resources in spatial locations could be improved^64^.

## Conclusions

Converging evidence indicates that people’s valuation of the environment is accomplished by running an internal model of the body in the world^12,13^. Such a model reflects people’s psychophysiological condition, based on past experiences and predicted needs^12–15^. Under this model, the main goal of the subjective valuation of the environment is the attainment of psychophysiological equilibrium^24^. To achieve this, people probably weigh bodily representations against value computations^22,23^, namely, how we feel and what the environment can provide. Yet, studies in social cartography usually neglect such evidence. Our study integrates what current studies in human psychophysiology often overlook (i.e., one’s ecological niche, beyond arbitrary stimuli in laboratory settings) with what studies in social cartography usually neglect (i.e., psychophysiological traits need to be accounted because they modulate peoples’ appraisals). Accordingly, we included in our study measures of affect and interoceptive sensibility. This offers a clearer and more complete understanding of human cognition in space.

Our new method and results show emotional memories and desired locations are consistently mapped in geographical space; that participants’ mapping correlates with their levels of stress, anxiety, and depression, as well as of interoceptive sensibility; and that natural environments relate more to positive memories, better psychological state, and interoceptive sensibility. We conclude that positive memories, desire to visit future locations (after the national Covid-19 lockdown), affect, and bodily sensibility are associated with discrete, yet partially overlapping, locations in space. Importantly, these locations were associated primarily with natural environments. Given that data was collected during a nationwide Covid-19 lockdown, our results support the idea that in ‘times of despair’ natural environments could favor psychological and physiological restoration^65,66^ by linking bodily representations to value computations^13,22,23,67^. Our results are also in line with contemporary models of allostasis by considering the affective, environmental, and bodily dimensions of human affect in topographical space. By revealing spatial maps of human cognition and linking these to individual differences, we provide the basis for the development of spatially contextualized markers of physical and mental disorders.

## Methods

### Participants

A total of 201 participants (69 males, 132 females, mean age = 32yo, age range = 18–66yo) completed the online study that included two self-report questionnaires and a novel geographical self-report task. Participants were recruited through opportunity sampling via the departments of Psychology of the University of the Balearic Islands, as well as through the local news. Ethical approval was granted by the Research Ethics Committee of the Balearic Islands. All procedures were conducted following the Declaration of Helsinki and informed consent was obtained from all participants. Participants for which questionnaire or demographic data were missing were excluded (33 of 234 participants, 14%).

### Task procedure

All participants performed the whole task procedure during the Spanish national Covid-19 lockdown (15th March–21st June 2020; data acquisition: 13^th^–26th April). First, participants completed two self-report questionnaires through the online platform Paperform: the Depression, Anxiety, and Stress Scale (Dass21^33,68^) and the Multidimensional Assessment of Interoceptive Awareness (i.e. MAIA^35,36^)—by using validated Spanish versions of the questionnaires^45,46^. The Dass21 measures negative affect associated with dysphoria and self-deprecation, autonomic arousal and experience of anxious affect, irritability and agitation. The MAIA measures 8 subscales that include the capability to notice, attend, regulate, listen, connect, and trust bodily sensations, as well as to ignore sensations of pain or discomfort.

Second, participants completed a geographical self-report task in which they had to shade within the outline of four identical blank maps (1) where they live, (2) where they had past positive and (3) negative experiences (happy and sad memories), and (4) where they most looked forward to going after the national Covid-19 lockdown. To do this, the participants downloaded an image file that included four maps with the corresponding indications above each map. Then, they shaded the maps by using a freely available online editor according to the instructions (Supp. Materials Fig. 1). Finally, they attached the resulting image to the end of the online study. The maps did not contain pointers, the painting was dynamic, thus successive strokes on a region increased the opacity of the paint (achieved by selecting lower opacity of the painting tool). More opacity corresponded to greater emotional intensity and the diameter of the painting tool was ~ 16 pixels. Finished images were stored in matrices where the paint intensity ranged from 0 to 1. Each map was represented by 63,741 pixels.

### Use of emotion categories and self-reports in the current task

As detailed above, in the first part of the study we asked people to assess their topographical space as a function of emotional memories. This process relies on accessing autobiographical memories associated with specific locations. When accessing autobiographical memories, it is common to assess past experiences by using emotional labels such as happiness and sadness^28–31^. Furthermore, we were interested in a general assessment of space using this kind of ‘general and basic’ emotions. At the implementation of a new method, these might provide more general information about space than complex emotions such as safety, envy, etc.

Aside from that, our study is based on the use of self-reports. The conclusions that can be drawn from the results need to be put into perspective. Self-reports provide abundant information but are also prone to measurement error^69^. For instance, participants might not be able to introspect or interpret the questions about interoceptive sensibility, or they could rate their affect better than it was during the data acquisition (i.e., desirability bias). In the context of the current study, further research should follow up self-reports with other methods^5,63^ across different timescales. In our case, for instance, by examining if desired locations in space were visited after the lockdown and/or using more objective measures of interoception.

### Preprocessing of data and statistical analysis

Data were screened manually and excluded for anomalous painting behaviour (e.g. drawing symbols). The geographical maps were pre-processed using a MATLAB (version R2018a) pipeline that was developed in-house. The image file with the four painted maps was split into four different matrices. All areas outside the boundaries of the maps were masked, such that pixels outside of the map could be discarded. The participants’ maps were analysed in three different ways. First, subject-wise emotional maps were created by subtracting the maps in which participants reported where they have experienced happy and sad memories. This allowed creating a continuous scale of positive–negative values associated with human emotional valence in geographical space. The corresponding maps were subjected to min–max normalization and smoothing of the data via 2D Gaussian smoothing kernel (as implemented in Matlab; *Imgaussfilt* function; sigma 1). Next, the emotional-spatial maps was subjected to Statistical Parametric Mapping (SPM12^43^) a statical technique used to examine brain activity recorded in functional neuroimaging experiments. In this context, we examined shaded pixels instead of voxels in the brain to quantify where in the maps and how strong different emotions relate to one’s geographical space (i.e. are locations consistently associated with particular emotional memories?); see Fig. 1C. Relatedly, it is worth noting that the current method is inspired by the observation/method that emotions can be mapped on the body by using a topographical self-report measure^70–72^.

Second, we examined whether certain areas of the emotional-spatial maps correlated with the participants’ scores in the Dass21 and MAIA self-report questionnaires (i.e. are geographical locations related to one’s affect?). To this aim, we subjected both the emotional-spatial maps and the scores of the questionnaires to SPM analysis. Also, we examined the properties of the areas revealed in the above analysis. Specifically, we estimated whether the areas revealed in the two previous analyses corresponded to natural or non-natural locations (i.e., do emotions and affect relate to specific environments?). To this end, we contrasted the subject-wise emotional-spatial maps, as well as the maps where participants reported where they wanted to go after the Spanish nationwide Covid-19 lockdown with publicly available governmental maps (Supp. Materials Fig. 2).

Then, we quantified the number of pixels overlapping with each environment and normalized these according to the total number of pixels existing in natural and non-natural environments. Non-natural areas comprised urban, crop and cattle raising areas, which are not usually available to the public and are artificially altered (therefore, non-natural areas could include patches of rural areas). The subsequent normalized proportions of natural and non-natural shaded pixels were subjected to repeated-measures ANOVA and follow-up with Paired Samples T-test, in case of deviation from normality Wilcoxon signed-rank was used. The corresponding data were plotted by using the Raincloud plots package in R^73^.

Third, we also estimated whether the participants lived in a more natural or non-natural location by computing the number of pixels in the participants living location that overlapped with these environments. Next, we examined if this index influenced how they related to past emotional experiences and their desire to visit further environments after the Covid-19 lockdown.

## Supplementary Information

Supplementary Information 1.

## Data Availability

We used in-house scripts to mask, smooth, and normalise the maps, as well as to compute the pixel quantification with overlapping environments. The code and anonymized data will be available upon publication in Open Science Framework (https://osf.io).
