# Supplementary material for: Emotional representations of space vary as a function of peoples’ affect and interoceptive sensibility
Source: Sci Rep. 2021 Aug 9;11:16150. doi: 10.1038/s41598-021-95081-9 (PMC8352937; doi:10.1038/s41598-021-95081-9)
Supplement: Supplementary file 1 — Supplementary Information 1. [file 41598_2021_95081_MOESM1_ESM.docx]

**Emotional representations of space vary as a function of peoples’
affect and interoceptive sensibility (*Supplementary materials*)**Alejandro Galvez-Pol, Marcos Nadal, and James M Kilner


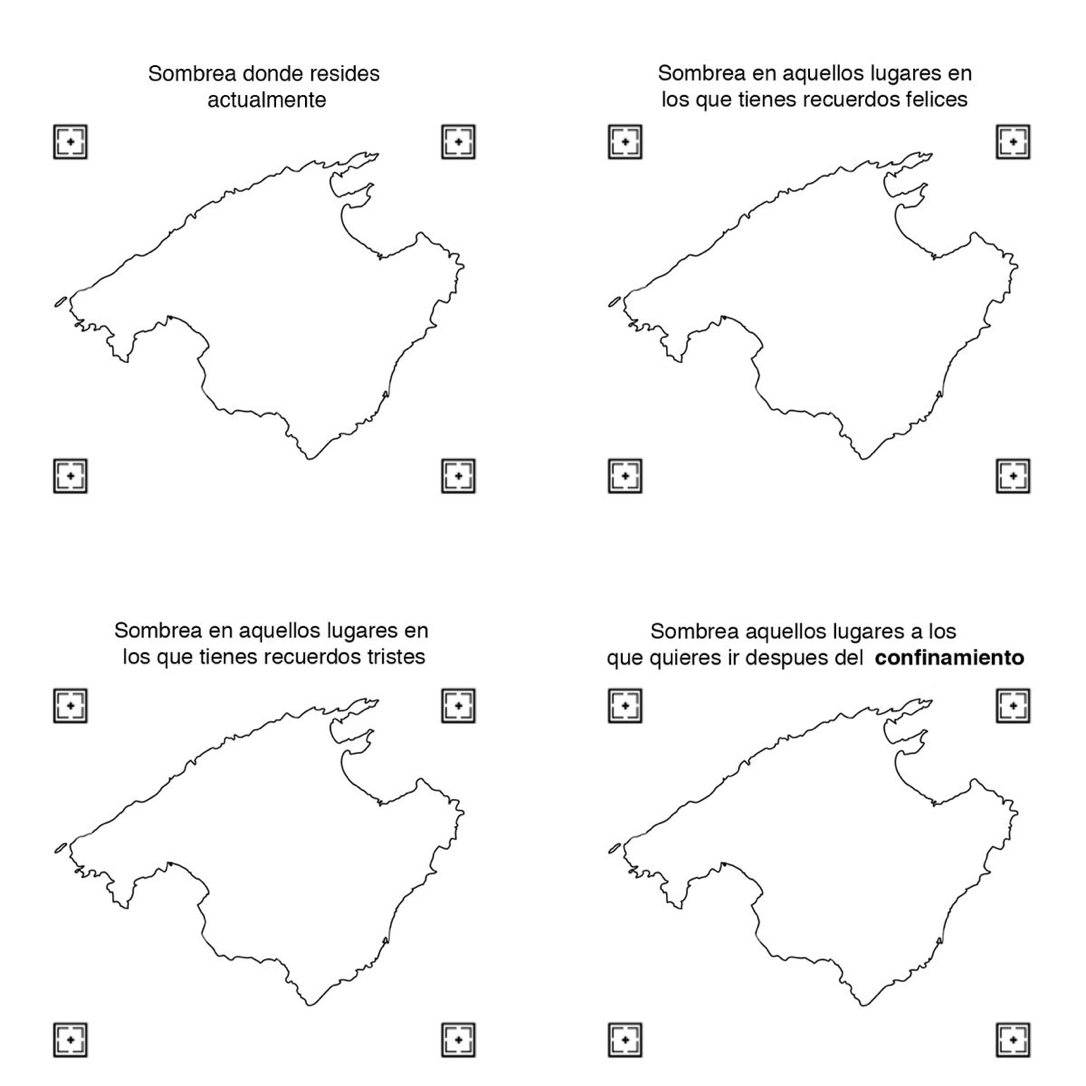


**Supp Fig. 1.** Participants completed a geographical self-report task in which they had to shade within the outline of four identical maps i) where they live, ii) where they had happy and iii) sad memories, and iv) where they most looked forward going to after the national Covid-19 lockdown; these indications are found in Spanish above each map. The participants downloaded the above image file after filling two self-report questionnaires and shaded in the maps by using a freely available painting tool (https://www.youidraw.com/apps/painter/).

**
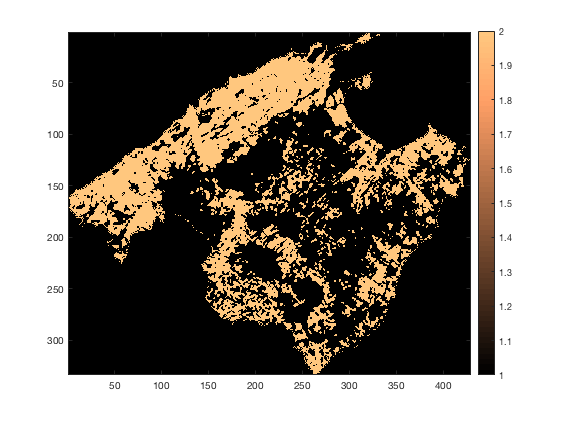
**

**Supp. Fig. 2. Mask used to compute the ratio of shaded pixels per environment**. We contrasted the subjectwise spatial maps, as well as where participants most looked forward going to after the nationwide Covid-19 lockdown with publicly available governmental maps (http://www.caib.es/sites/sanitatforestal/es/n/mapa_forestal_de_mallorca-31740/). Then, we converted this map into a binary mask where 0 denoted green spaces and natural regions and 1 non-natural regions (the latter could comprise urban and crop regions). Then, we quantified the number of pixels shaded by the participants that overlapped with each environment and normalized these according to the total number of pixels existing in the two environments.

**
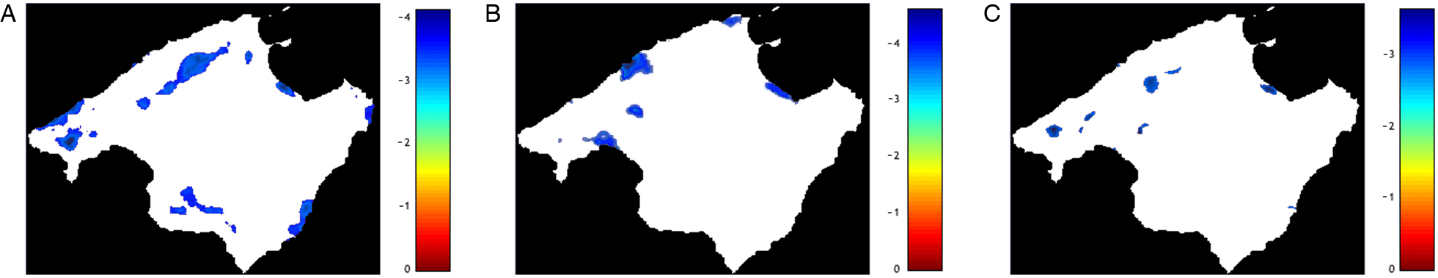
**

**Supp. Fig. 3. Spatial maps of emotions correlated with subscales of the Dass21 questionnaire**. We examined the relationship between participants’ affect and geographical space by correlating their scores in the Dass21 questionnaire with their maps. The correlation of each subscale of the Dass21 (depression, anxiety, and stress) revealed smaller regional clusters that negatively correlated with each subscale (panels A, B, and C, respectively; uncorr. in SPM at 0.01); n=201; colorbars denote the t-statistic range.

**
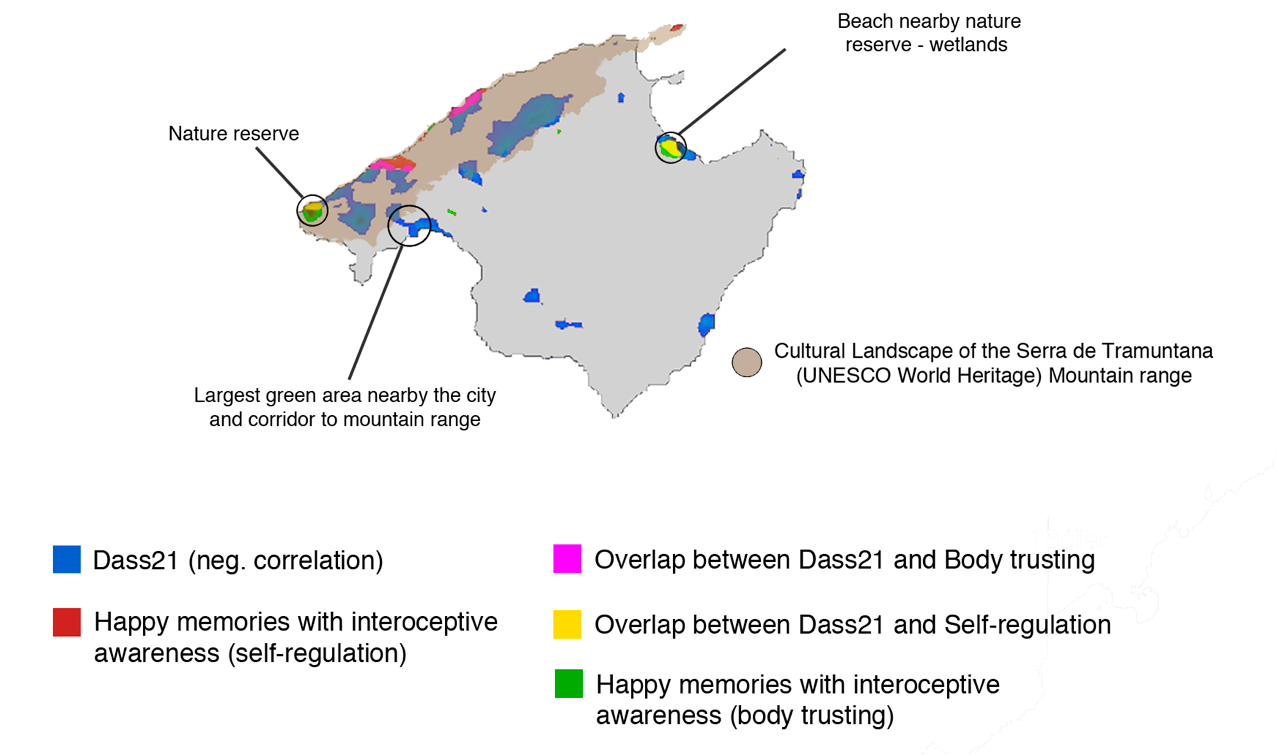
**

**Supp. Fig. 4. Circumscribed green and natural spaces overlap with spatial correlates of happy memories in the territory.** Overlap between significant clusters in the SPM analysis and protected natural and green spaces. Most of the significant clusters that correlated with participants’ affect (negative correlation with Dass21) were found along the mountain range Serra de Tramuntana; UNESCO World Heritage. Other regions included the largest green area nearby the city of Palma de Mallorca (capital of the island) and coastal sites.

**Supplementary analysis.** we computed in a single value whether participants reported more overall memories in natural or non-natural areas, i.e., regardless of the valence, an index based on subtracting the proportions of shaded pixels in natural and non-natural areas. Then we correlated this index with participants’ scores on the Dass21, and on the subscales Self-Regulation and Body Trusting of the MAIA questionnaire. The results showed significant correlations with these scores. Participants who reported a greater proportion of natural areas also reported better affect (rs = -0.175, *p* = 0.01), more sensibility to regulate distress by attending to bodily sensation, as well as the experience of one’s body as safe (rs = 0.216, *p* = 0.002; rs = 0.165, *p* = 0.019, respectively).

Likewise, we also computed in a single value participants’ desired environment after the nationwide Covid-19 lockdown and correlated this index with the participants’ scores on the Dass21, and on the subscales Self-Regulation and Body Trusting of the MAIA questionnaire. The results showed a significant correlation between these scores and participants’ desire for natural locations. Participants who reported a greater proportion of natural areas reported better affect (negative correlation with Dass21; rs = -0.179, *p* = 0.01), better regulation of distress by attending to bodily sensations, and experience of their body as safer (rs = 0.214, *p* = 0.002; rs = 0.193, *p* = 0.006; Supp. Fig 5 below).


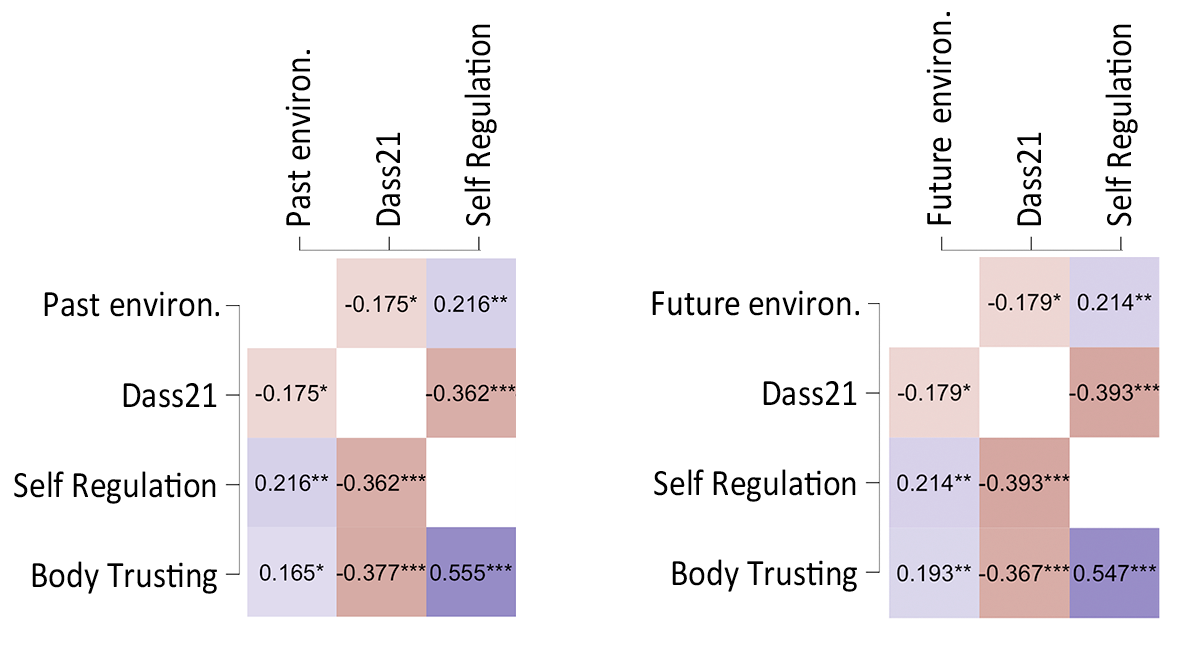


**Supp. Fig. 5. Correlations between environment, Dass21, and MAIA.** The heatmap displays Spearman’s rho correlations. These revealed that higher levels of depression, anxiety, and stress (as measured by the Dass21) negatively correlated with shading greater proportions of natural environment in both emotional memories (Past environment) and future desired locations (Future environment). The opposite pattern was found for the subscales Self-regulation and Body Trusting of the MAIA questionnaire. Higher scores in these dimensions of interoceptive sensibility correlated with shading greater proportions of natural environment.

**
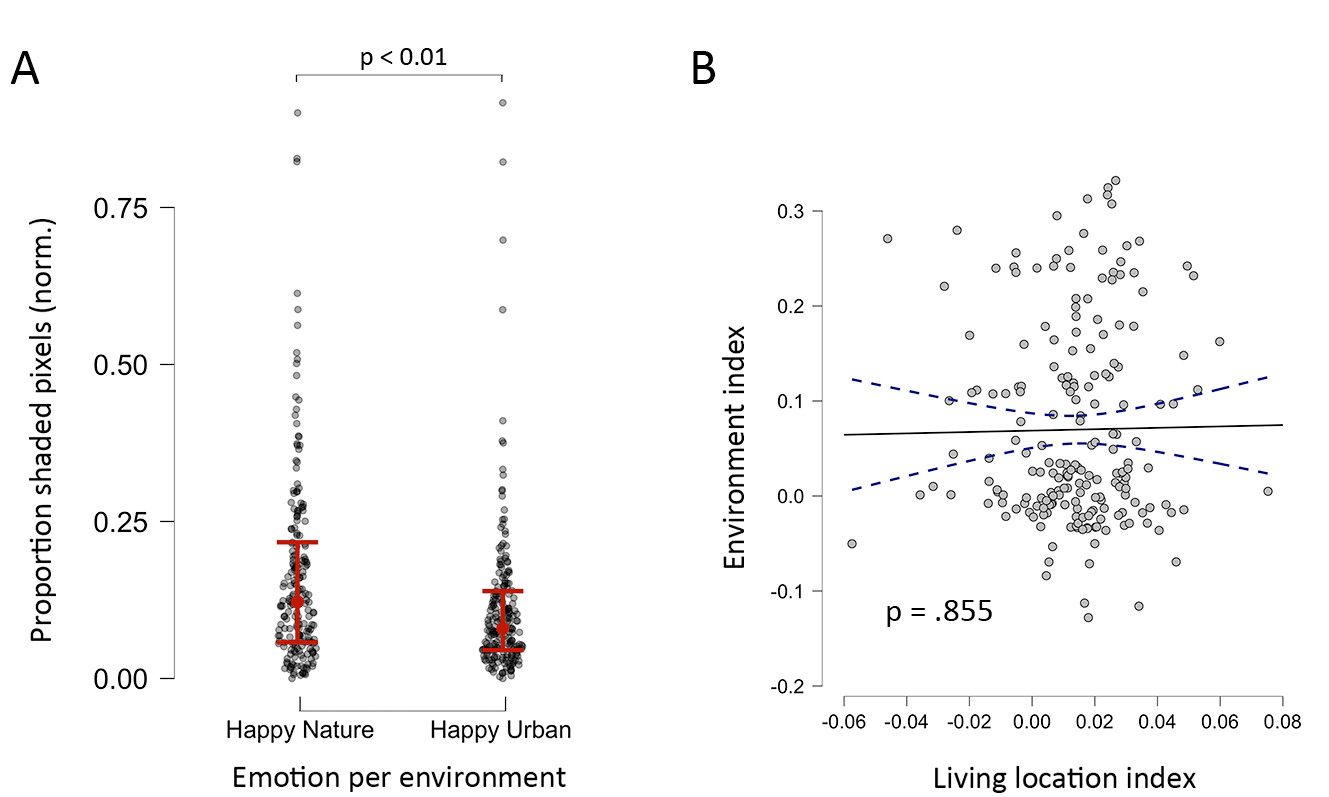
**

**Supp. Fig. 6. Living location does not moderate environmental preference.** (A) We mitigated the effect of participants’ living location in the maps of emotional memories by subtracting participants’ living location maps from their subject-wise emotional maps (see main results and methods). Then, we renormalised the proportions of shaded pixels per environment in a participant basis. The results did not change, participants reported more happy memories in natural areas (*p* < 0.01). (B) We also correlated participants’ living location and where they wanted to go after the nationwide lockdown. To this end, we computed an ‘environmental index’ by subtracting the number of shaded pixels in non-natural locations to those in natural locations. Likewise, we computed a ‘living location index’ by subtracting the number of shaded pixels in natural regions to non-natural regions of the participants’ living location maps. The results showed that living location did not predict the environmental preference after lockdown (*F*_(1,199)_ = 0.034, *p* = .855), R^2^ = -0.005. n=201; positive values in the Environmental index denote desire for natural and green locations; positive values in the Living location index denote that most participants lived in urban regions.

**
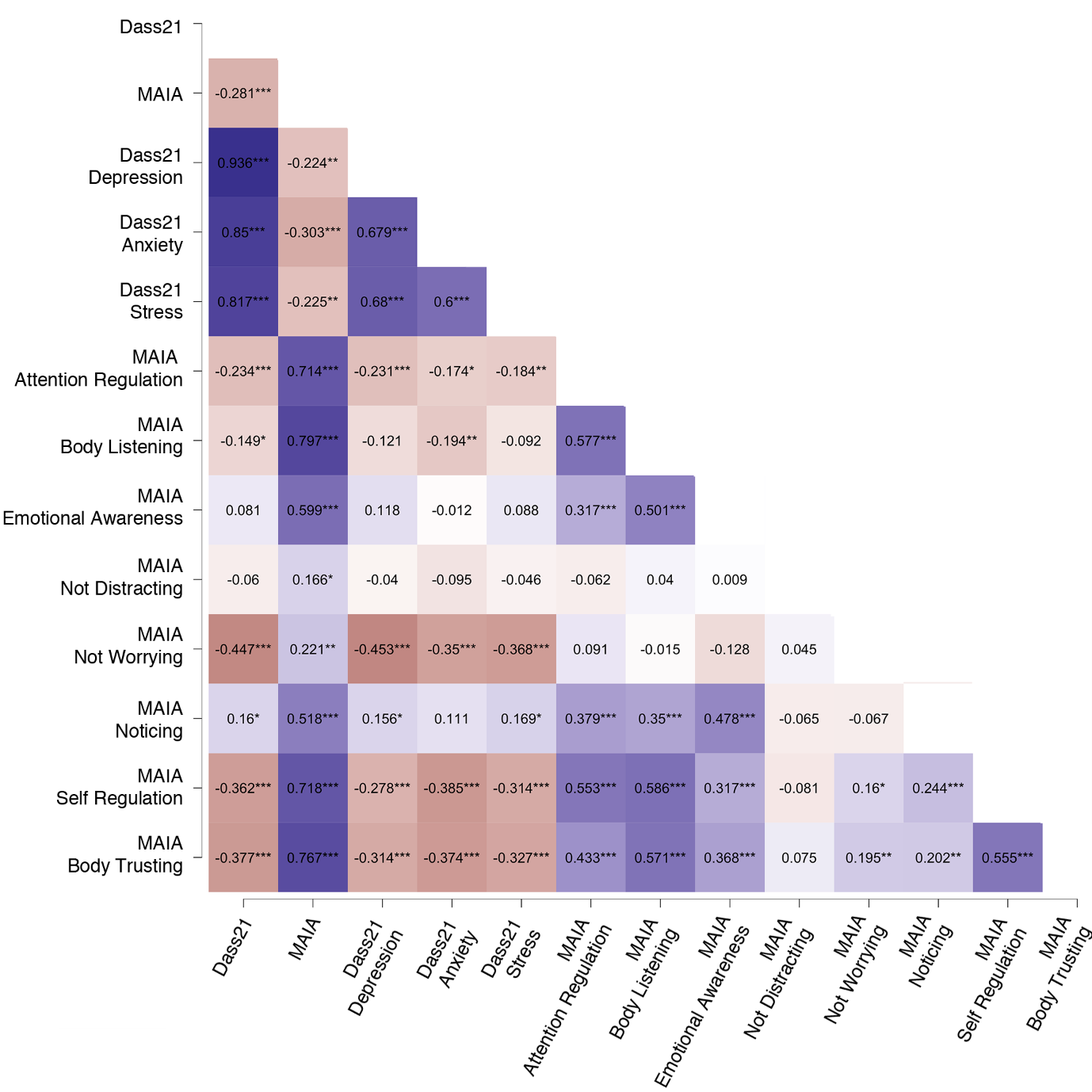
Supp. Fig. 7. Correlations between Dass21 and MAIA including subscales.** The heatmap displays Spearman’s rho correlations. Overall, these revealed that higher levels of depression, anxiety, and stress (as measured by the Dass21) correlated negatively with interoceptive sensibility and subsequent subscales (as measured by the MAIA).
